# The effect of three different insoles on balance in people with functional ankle instability

**DOI:** 10.1186/1757-1146-7-S1-A19

**Published:** 2014-04-08

**Authors:** Khadijeh Bapirzadeh, Akram Jamali, Saeed Forghany, Christopher Nester, Sanam Tavakoli, Fateme Hemmati

**Affiliations:** 1 Musculoskeletal Research Centre, Isfahan University of Medical Sciences, Iran; 2 Centre for Health Sciences Research, University of Salford, UK

## Background

Functional ankle instability (FAI) has been reported to be associated with sensorimotor deficits which could result in impaired balance [1] and altered foot kinematics [2]. Textured insoles that increase plantar cutaneous afferent information may compensate for a deficit of sensory input in FAI, improving postural control and reduce the risk of injury. Also, a lateral plantar wedge will reduce the external ankle inversion moment and help prevent inappropriate foot inversion motion and perhaps further improve postural control. The aim of this study therefore was to investigate the effect of texture and a lateral wedge on standing balance in people with FAI.

## Methods

20 athletes (age: 26.55±5.35years) with clinically diagnosed FAI were recruited. Static balance in double limb stance was assessed using Kistler force plates during four shod conditions: 1) flat EVA base insole 2) Textured flat EVA insole 3) Lateral heel and sole wedge (Salford insole) 4) Textured lateral heel and sole wedge (Salford insole). Texture was a semirigid rubber with semi-circular mounds with center to center distances of 4 mm. The center-of-pressure excursion and mean velocity in anterior-posterior and medial-lateral directions and area of 95% confidence circle were derived as measures of standing balance. The results were statistically analyzed using the nonparametric Fridman test followed by Wilcoxon Signed Rank.

## Result

Statistically significant differences were observed only for the textured flat EVA insole. The mean COP velocity was reduced compared to the lateral wedge condition (p <0.05) and the 95% confidence circle area decreased significantly compared with all other insoles conditions (Table 1).There were no statistically significant effects from the lateral wedge.

**Table 1:** Mean COP parameters during different insole conditions

		F ^a^	TF ^b^	L ^c^	TL ^d^
Mean COP excursion (mm)	ML ^e^	5.04±1.68	4.81±1.56	5.29±2.1	5.41±2.27
	AP ^f^	3.02±0.91	2.75±0.89	2.92±1.29	2.74±1.06
	TOTAL	6.59±1.98	6.13±1.96	6.49±2.18	6.35±2.21
Mean COP velocity (mms^-1^)	ML	7.87±2.25	7.69±2.24	8.23±1.9	7.99±2.4
	AP	5.66±2.56	5.32±2.32 (L^**^)	5.9±1.96	5.39±2.17
	TOTAL	11.1±4.2	10.1±2.87	11.2±2.77	10.5±3.49
95% confidence circle area		621.44±440.03	433.99±243.87 (L^**^,F^*^,TL^*^)	608.89±355.17	613.46±539.88

^a^ Flat EVA. ^b^ Textured flat EVA. ^c^ lateral wedge. ^d^ Textured lateral wedge. ^e^ Medial-Lateral. ^f^ Anterior-Posterior. ^*^ P <0.05. ^**^ P <0.001.

## Conclusion

Texture appears to have some impact on standing balance but only on a flat insole. The lateral wedge had no effect on standing balance.

## Competing interests

Nester declares a personal commercial interest in the insoles tested in this study.
